# Do larger genomes contain more diverse transposable elements?

**DOI:** 10.1186/s12862-015-0339-8

**Published:** 2015-04-22

**Authors:** Tyler A Elliott, T Ryan Gregory

**Affiliations:** Department of Integrative Biology, University of Guelph, 50 Stone Road East, Guelph, Ontario N1G 2W1 Canada

**Keywords:** C-value, DNA transposon, Genome sequencing, LTR retrotransposon, LINE, SINE

## Abstract

**Background:**

The genomes of eukaryotes vary enormously in size, with much of this diversity driven by differences in the abundances of transposable elements (TEs). There is also substantial structural and phylogenetic diversity among TEs, such that they can be classified into distinct classes, superfamilies, and families. Possible relationships between TE diversity (and not just abundance) and genome size have not been investigated to date, though there are reasons to expect either a positive or a negative correlation. This study compares data from 257 species of animals, plants, fungi, and “protists” to determine whether TE diversity at the superfamily level is related to genome size.

**Results:**

No simple relationship was found between TE diversity and genome size. There is no significant correlation across all eukaryotes, but there is a positive correlation for genomes below 500Mbp and a negative correlation among land plants. No relationships were found across animals or within vertebrates. Some TE superfamilies tend to be present across all major groups of eukaryotes, but there is considerable variance in TE diversity in different taxa.

**Conclusions:**

Differences in genome size are thought to arise primarily through accumulation of TEs, but beyond a certain point (~500 Mbp), TE diversity does not increase with genome size. Several possible explanations for these complex patterns are discussed, and recommendations to facilitate future analyses are provided.

**Electronic supplementary material:**

The online version of this article (doi:10.1186/s12862-015-0339-8) contains supplementary material, which is available to authorized users.

## Background

The genomes of Bacteria and Archaea are generally quite small and their sizes are determined in large measure by the number of protein-coding genes that they contain [1]. The situation is very different for Eukaryotes, in which nuclear genome size estimates range nearly 70,000-fold, from a mere 2.3 megabase pairs (Mbp) in the microsporidean parasite *Encephalitozoon intestinalis* to an astounding 148,852 Mbp in the lilly *Paris japonica* [2,3]. Even within taxa, genome sizes range dramatically: more than 7,000-fold among animals (and 350-fold among vertebrates alone) [4] and 2,400-fold across land plants [5]. In Eukaryotes, genome size diversity among taxa is largely unrelated to number of protein-coding genes, and instead is determined primarily by substantial differences in the quantity of non-coding DNA. In particular, transposable elements (TEs) appear to be represent of the dominant contributors to overall genome size variability among Eukaryotes [6,7].

TEs are divided into two major classes according to whether they employ an RNA intermediate in a copy-and-paste mechanism of transposition (Class I, or retrotransposons) or transpose via a direct cut-and-paste mode without reverse transcription (Class II, or DNA transposons). Within each class, TEs are further classified into orders, superfamilies, and families based on shared structural features and overall sequence similarity [8,9]. As such, it is possible to examine not only TE abundance, but also TE diversity – i.e., the distinct number of TE taxa, such as superfamilies – within and among eukaryotic genomes of different sizes.

On the face of it, one might expect larger genomes to contain more types of TEs as well as more TE copies than smaller genomes. For example, the yeast *Saccharomyces cerevisiae* has a tiny genome (~12 Mbp) whose constituent TEs include only members of the *Gypsy* and *Copia* long-terminal repeat (LTR) retrotransposon superfamilies [10]. By contrast, the much larger human genome (3,200 Mbp) contains not only a large abundance of particular TEs (over a million copies of the short interspersed nuclear element (SINE) *Alu*, for example), but also a substantial number of TE superfamilies and their extinct remnants [11]. A general relationship between TE diversity and genome size would reveal itself as a positive correlation between the two parameters, though perhaps one that levels off at a certain point as the available diversity of TEs is exhausted and genomes become saturated with the different types of TEs.

On the other hand, it has been pointed out that pufferfishes exhibit much higher TE diversity and many more active TE families than humans, despite having genomes only one tenth as large [12]. This latter observation has led to the suggestion that smaller genomes may, in fact, harbour a greater diversity of TEs, perhaps because intense competition among TEs for limited insertion sites and/or host-parasite coevolution with the genome’s deletion mechanisms leading to diversification at the TE level [7]. At present, the most that can be said is that it remains unclear what relationship exists between genome size and TE diversity (if any), because the issue has never been examined in detail.

Here, a compiled dataset of sequenced genomes is used to evaluate possible correlations between genome size and TE diversity. In the process, two opposing hypotheses are tested: 1) whether genomic expansion is driven by, or at least associated with, an increase in TE diversity as well as TE abundance, or 2) whether the initial comparison of pufferfish and human holds more broadly (and if so, at what scales), such that constraints on genome size actually drive diversification of TEs and/or promote the coexistence of more diverse TEs.

## Results

### Patterns across eukaryotes

**Figure 1:**
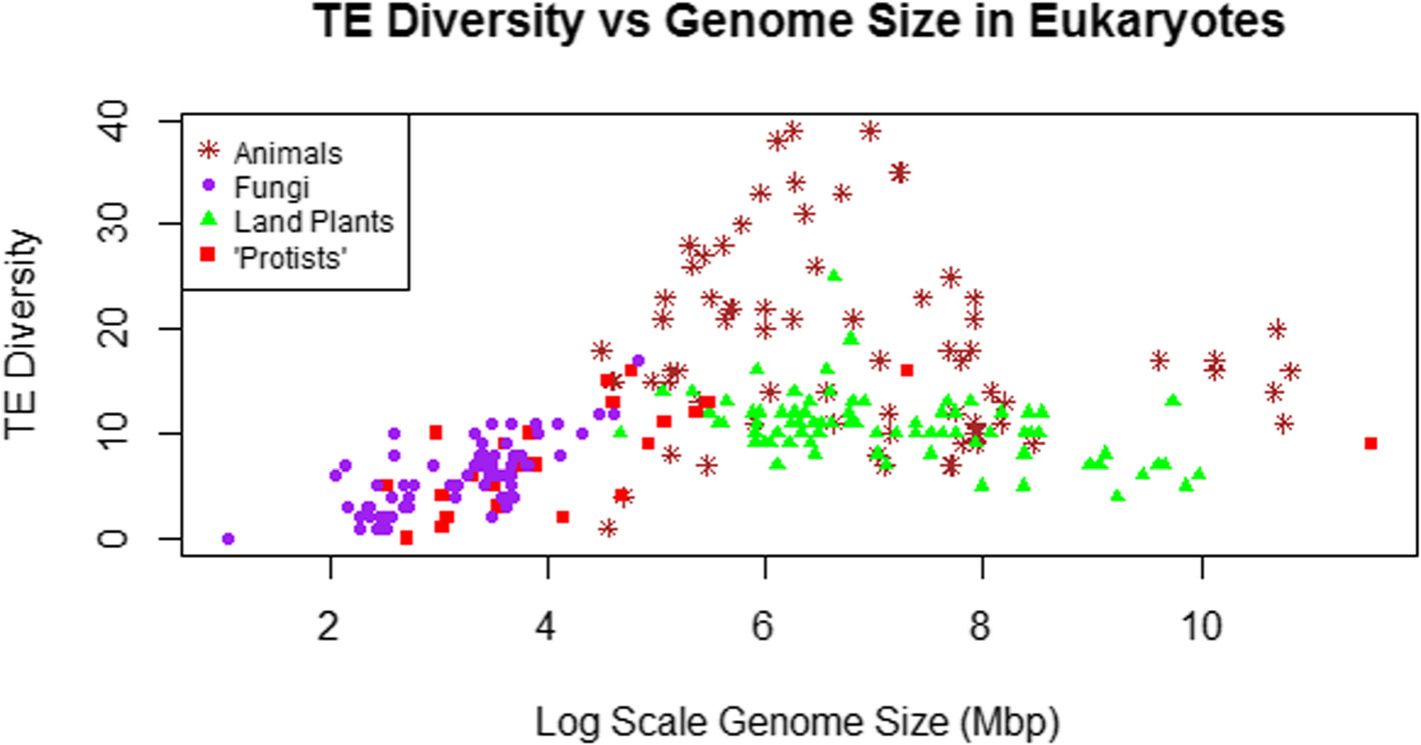
Number of superfamilies (TE diversity) and log-scale genome size (Mbp) in 257 eukaryote genomes. Brown points represent animal genomes, green points represent land plant genomes, purple points represent fungal genomes and red points represent “protist” genomes. This includes all available data, regardless of TE discovery and annotation method (cf. Figure 6).

**Figure 2:**
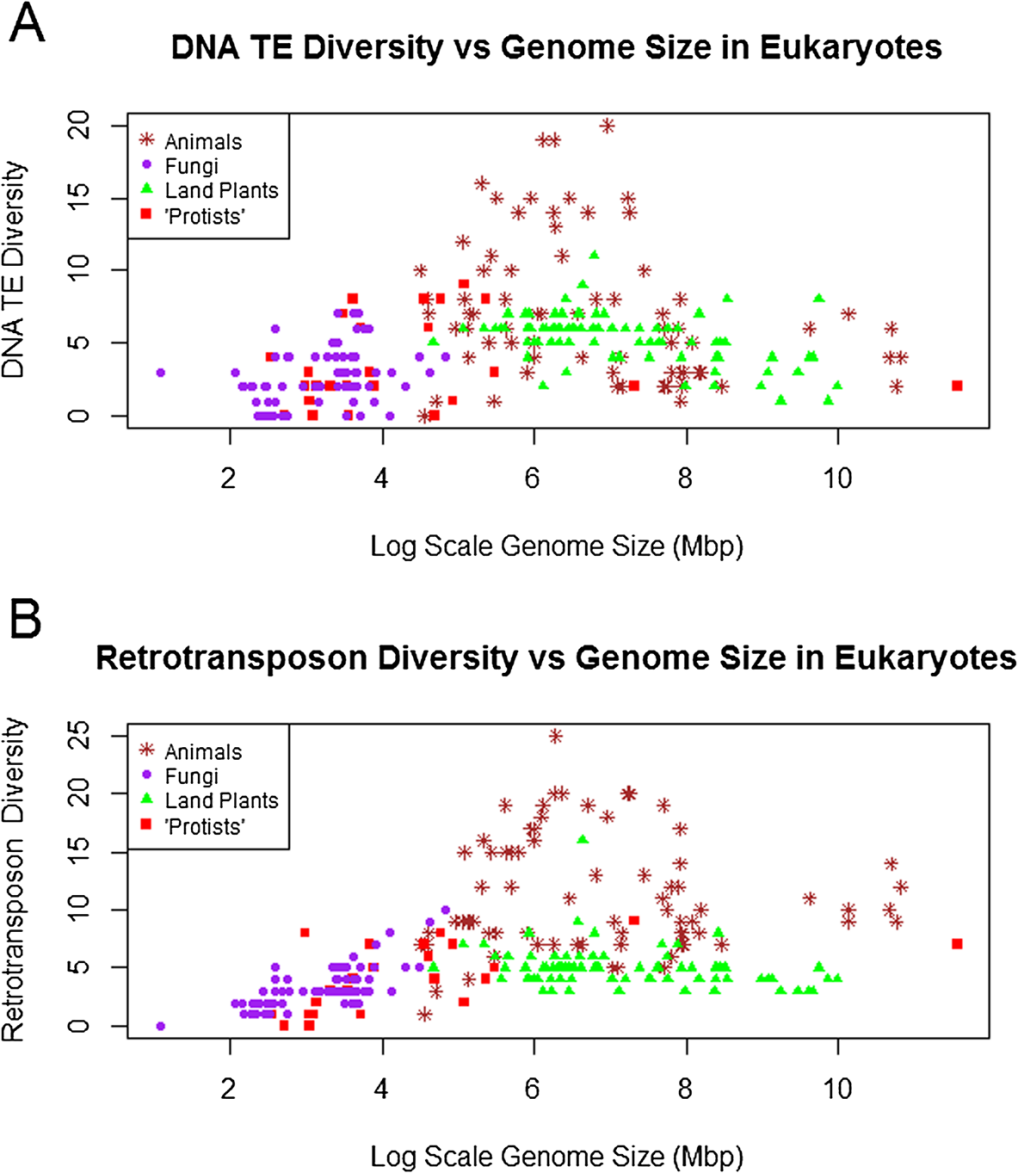
TE diversity versus genome size separated into the two TE classes. **(A)** Number of superfamilies’ (TE diversity) of DNA transposons and log-scale genome size (Mbp) in 257 eukaryote genomes. **(B)** Number of superfamilies’ (TE diversity) of retrotransposons and log-scale genome size (Mbp) in 257 eukaryote genomes. Brown points represent animal genomes, green points represent land plant genomes, purple points represent fungal genomes and red points represent “protist’ genomes.

Overall, there was no linear relationship between diversity of TE superfamilies and genome size when all eukaryote data were included (r = 0.04, p > 0.5, n = 257). As shown in Figure 1, the relationship is more complex and leads to a bell-shaped distribution, with comparatively low TE diversity found in both small (<100 Mbp) and large (>2,000 Mbp) genomes but a wide range of total TE superfamily diversity observed in mid-sized genomes (~100 Mbp to 2,000 Mbp). Maximum TE diversity (39 superfamilies present) occurs in genomes around 500Mbp in size. Similar patterns were observed for both Class I (retrotransposons) and Class II (DNA transposons) taken separately (Figure 2).

### Patterns in specific taxa

**Figure 3:**
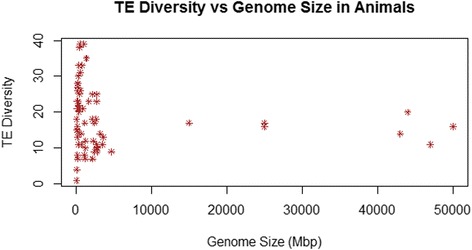
Number of superfamilies (TE diversity) and genome size (Mbp) in 75 animal genomes. There was no linear relationship across all animals (r = −0.12, p > 0.3).

**Figure 4:**
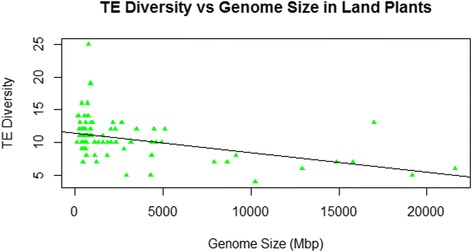
Number of superfamilies (TE diversity) and genome size (Mbp) in 80 land plant genomes. The line represents the significant negative correlation between TE diversity and genome size among plants (r = −0.44, p < 0.0001).

**Figure 5:**
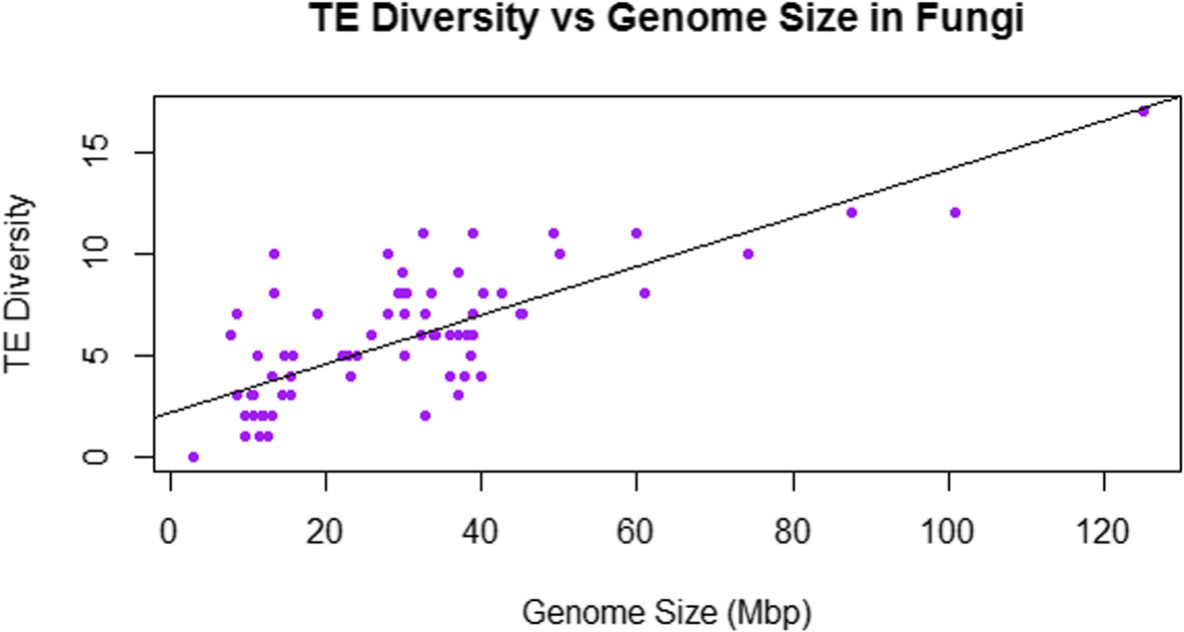
TE diversity versus genome size in fungi. Number of superfamilies (TE diversity) and genome size (Mbp) in 77 fungal genomes. The line represents the significant positive correlation between TE diversity and genome size in fungi (r = 0.764, p < 0.0001).

As is apparent in Figures 1 and 2, there is substantial taxonomic clustering of the data, with most of the data for smaller genomes coming from fungi and “protists” and the larger genomes belonging to animals and land plants. For this reason, analyses of TE diversity versus genome size were also conducted within individual taxonomic groups. There was no linear relationship within vertebrates (r = 0.03, p > 0.86, n = 34) nor among all animals (r = −0.12, p > 0.3, n = 75) (Figure 3). However, a significant negative correlation was found within land plants (r = −0.44, p < 0.0001, n = 80), which persisted following phylogenetic correction (r = −0.306, p < 0.006, n = 79 contrasts). As shown in Figure 4, the land plant data are characterized by high variance in TE diversity at smaller genome sizes and exclusively low diversity in large genomes. By contrast, there was a significant positive correlation within fungi, which again was significant without phylogenetic correction (r = 0.764, p < 0.0001, n = 77; see Figure 5) or when phylogenetically independent contrasts (PICs) were used (r = 0.649, p < 0.0001, n = 76 contrasts).

### Patterns according to genome size range

The distribution pictured in Figure 1 suggested that there could be two distinct relationships between TE diversity and genome size: a positive correlation among genome sizes of less than about 500Mbp and a negative correlation above this hypothetical turning point. When all available eukaryotes with genome sizes <500 Mbp were analyzed, a significant positive relationship was observed (r = 0.63, p < 0.0001, n = 150), including after phylogenetic correction (r = 0.357, p < 0.0001, n = 145 contrasts). However, when all genomes >500Mbp were analyzed together, no significant correlation was found (r = −0.09, p > 0.35, n = 107).

### Patterns of TE superfamily distribution across genomes

In general, animals exhibited the greatest variance in reported TE diversity, ranging from one superfamily in the canine heartworm, *Dirofilaria immitis*, to a maximum of 39 superfamilies in the genomes of *Branchiostoma floridae* (lancelet; 1C = 520Mbp), *Bombyx mori* (silkworm moth; 1C = 530Mbp), and *Hydra magnipapillata* (freshwater hydra; 1C = 1050Mbp) (Table 1). Despite also possessing a significant range in genome sizes, land plants displayed much lower overall variability in TE diversity as compared to animals. Fungi and protists had the lowest average TE diversity and the smallest total genome sizes. It should be noted that the superfamily count tabulated for each genome may be underestimated, especially in less well-studied genomes; however this is not expected to affect the overall patterns observed.

**Table 1:** **Summary statistics for TE diversity (number of superfamilies) in each of the taxonomic groups studied**

	**Animals**	**Vertebrates only**	**Land plants**	**Fungi**	**“Protists”**
**Mean**	18.32	14.91	10.53	5.66	7.96
**SD**	9.04	6.58	3.12	3.32	4.58
**Range**	38	28	25	17	16
**Variance**	81.79	43.36	9.75	11.02	20.96

A number of superfamilies were found to be present in all taxonomic groups examined, including common TE superfamilies such as Tc1/*Mariner*, *hAT*, *Gypsy*, and *Copia* (Table 2). In general, the superfamilies that were found in all of the major taxa also tended to be common among species within those taxonomic groups (Table 3). For example, *Gypsy* and *Copia* LTR retrotransposon superfamilies were found in every one of the plant genomes examined, and were also among the more abundant elements in at least some representatives of each of the other major taxa. Similarly, the *hAT* and Tc1/*Mariner* DNA transposon superfamilies were among the top five most abundant categories of TEs found in all groups examined. By contrast, non-LTR retrotransposon superfamilies (especially *CR1*, *L1*, and *RTE* elements) were only abundant within animals and “protists” and not in land plants or fungi.

**Table 2:** **TE superfamilies found in all taxonomic groups studied**

**Retrotransposons**	**DNA Transposons**
*Gypsy*, *Copia*, L1, RTE, CR1/L3, L2, R1, *Penelope*, SINE2 tRNA	Tc1/*Mariner*, *Merlin*, PIF/*Harbinger* + ISL2EU, *Mutator* + *Rehavkus*, P-element, *hAT*, *PiggyBac*, *CMC*, *Helitron*, *Maverick*/*Polinton*, *Crypton*

**Table 3:** **TE superfamilies found in only one taxonomic group**

**Animals**	**Fungi**	**“Protists”**
*Sola2*, *Academ*, *Zator*, *Zisupton*, IS3EU, IS4EU, *Crack*, *Nimb*, *Soliton*, *Proto1*, *Proto2*, *Hero*, LOA, *Outcast*, *Daphne*, L2A, L2B, *Vingi*, *Kiri*	*Tad1*	RTETP, *Ambal, Novosib*, *Dualen*/*RandI*(Green Algae)

### Effects of TE discovery method

**Figure 6:**
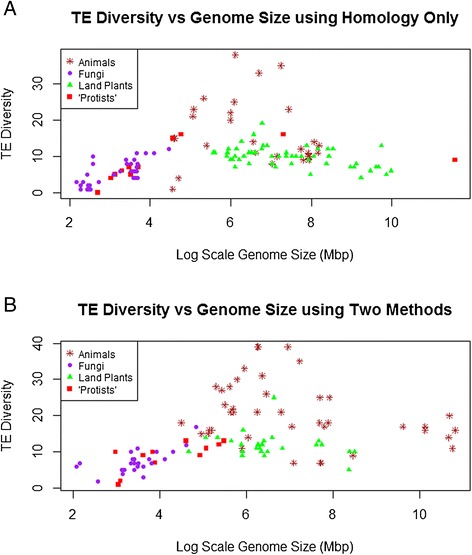
Effects of TE discovery method. The overall pattern of TE diversity versus genome size among eukaryotes according to whether TE discovery was **A)** based only on sequence similarity against an existing database or **B)** based on both sequence similarity and *de novo* discovery. (A much smaller number of studies used only *de novo* methods, and are not shown in a separate analysis). Importantly, the general patterns are the same regardless of TE discovery method(s) used (see also Figure 1). Brown points represent animal genomes, green points represent land plant genomes, purple points represent fungal genomes and red points represent “protist” genomes.

Two approaches are generally employed in the discovery and annotation of TEs in eukaryotic genomes: either identifying sequence similarity versus existing databases or finding potential TEs through *de novo* discovery of repeated elements. As shown in Figure 6, the overall pattern of TE diversity versus genome size is fundamentally similar to that shown in Figure 1 regardless of whether the TE data were generated using only sequence similarity or both of the available methods.

## Discussion

### TE diversity versus genome size

It would seem to be a straightforward expectation that larger genomes would contain both more types of TEs and more copies thereof than smaller genomes. However, the central finding of the present analysis is that any relationships between TE diversity and genome size are much more complex than this. No linear correlation was found across the full range of available genomes nor within vertebrates or among all animals. By contrast, there was a positive correlation among fungi and other species with genome sizes <500 Mbp but a negative correlation in land plants. This lack of any clear association between TE diversity and genome size indicates that eukaryotic genomes – at least those above a comparatively low threshold of ~500 Mbp – do not generally grow via the accumulation of an expanded array of TEs. Indeed, it seems that the largest animal and land plant genomes sequenced to date exhibit relatively depauperate TE diversity as compared to mid-sized genomes in these groups. By extension, the results of the present study suggest that TE abundance (total number of TEs of all types, a major determinant of genome size) and TE diversity (number of different types of TEs) are at least partially decoupled across eukaryotes.

The present findings raise the question of why TE diversity and genome size are positively correlated for genome sizes up to ~500 Mbp but not above this apparent threshold. An obvious possibility is that this pattern reflects taxonomic bias or other limitations of the available dataset. Most of the genomes below 500 Mbp in the current dataset are found in fungi and “protists”, whereas nearly all of the data for genomes larger than this were from animals and land plants. Moreover, 40% of the data from genomes >500 Mbp came from survey sequencing projects, raising the possibility that the TE diversity of larger genomes is underreported. These are unlikely to provide sufficient explanations, however, as the pattern in small genomes holds across distantly related fungi and protists and following phylogenetic correction. In addition, there is no apparent correlation between estimated TE diversity and either read length (p > 0.16) or depth of coverage (p > 0.25) among the survey sequenced genomes included in the present study.

Another explanation worth considering for the overall pattern is that genome expansion is initially driven by an increase in both TE abundance and TE diversity, but beyond a certain point TE diversity becomes saturated. That is, once a genome reaches about 500 Mbp in size, it already contains the complete set of the most common types of TEs and all that remains for further growth is an increase in abundance of those elements. This is likely to be a significant factor in explaining the positive correlation up to ~500 Mbp, but it does not account for the overall pattern reported here because TE diversity does not simply level off at the maximum as genome sizes increase beyond 500 Mbp. Rather, there is a substantial increase in total variance of TE diversity in mid-sized genomes, and a decrease in both variance and maximum TE diversity in the larger genomes of animals and land plants (and at least one protist).

Instead, the most plausible explanation is that TE diversity and abundance both increase as genome sizes expand up to a moderate size, whereas further genomic growth beyond this point is driven by a major surge in abundance of a small subset of initial TE diversity. In humans, for example, there are 14 TE superfamilies, but total TE content is heavily biased in favour of a small number of hyperabundant elements including >1 million copies of *Alu* and >500,000 copies of *LINE-1*, constituting approximately 322 Mbp and 533 Mbp of the assembled human genome respectively. However, only a few hundred copies of *LINE-*1 remain active, the remainder being inactive remnants [13,14]. In contrast, the 10-fold smaller genome of the pufferfish *Takifugu rubripes* contains about 20,000 TE copies in total, but 22 active, or recently active, superfamilies comprising only 23Mbp of its estimated genome size [15].

Theoretical investigations have suggested that stronger selection against the deleterious effects of TE insertions results in fewer copies, but also leads to a higher percentage of active elements [16-18]. Inactive (or less active) elements have a lower chance of surviving the selective filter that the host genome presents, possibly winnowing TE diversity over long time periods. In mid-sized genomes, host level selection limits copy number but also helps to maintain more active elements, which means more superfamilies of TEs that survive in the genome (albeit in low copy numbers). By contrast, in very small genomes there is insufficient real estate to accommodate a large diversity of active TE superfamilies.

Additional factors, such as horizontal transfer rates, host and transposable element demography, and competition between elements, probably contribute to the expansion of variance in TE diversity as one approaches the middle range of genome sizes [19,20]. Further, it has been suggested that selection pressure arising from ectopic recombination would engender a richer community of TEs as this would mitigate deleterious effects on the host and prevent removal of elements from the genome [21,12]. Abrusán and Krambeck [22] suggested that copy number and richness dynamics might hinge upon the strength of silencing mechanisms and the degree of cross reaction of said mechanisms across divergent types of TEs. Data from the seven genomes they compared matched well with these predictions, with two of the smallest genomes predicted to have strong sequence specific silencing, low element copy numbers and higher TE richness. Lack of knowledge about the specific mechanisms of silencing in a variety of taxa make this difficult to test on a broad scale, but it remains an interesting option to consider.

### Ubiquitous versus taxon-specific TE superfamilies

It appears that there is a much wider range in total TE diversity in medium-sized animal genomes as compared to those of land plants, which could explain why there is no significant negative correlation between TE diversity and genome size in animals as there is among plants. In turn, this probably relates to the greater number of animal-specific TE superfamilies that have been identified, such as *Zator*, *Soliton*, and others (Table 3). This difference between animals and plants in TE diversity may be real, or it may partially reflect differences in the phylogenetic diversity of the species for which data are available, being much broader in animals than in plants, or it could be the product of lower-resolution descriptions of TEs in sequenced plant genomes.

On the other hand, there are several groups of TEs that are found broadly across taxa. For example, both *Copia* and *Gypsy* LTR retrotransposons were top hits in all taxonomic categories (Table 4). Why these particular elements tend to be ubiquitous among eukaryotes awaits explanation, but there is some reason to expect LTR retrotransposons to be present more broadly than other element types. Within Class I elements, *Penelope* and LINE elements employ a target-primed reverse transcription system of replication which seems to make them more prone to creating dead-on-arrival inserts, which are 5’ truncated and less likely to be capable of another round of replication [23]. LTR elements are not known to do this, potentially leading to a higher proportion of new inserts that remain active and thus capable of creating additional copies in their turn. In addition, LTR elements are known to acquire *Env* open reading frames and appear to be more capable of horizontal transfer events than LINEs. However, this has been most readily observed in drosophilids; therefore whether this is a common phenomenon in LTR elements in other taxonomic groups is unknown at this time [20]. Notably, El Baidouri et al. [24] recently reported evidence of frequent horizontal transfer of LTR retrotransposons in a survey of 40 plant genomes. Within Class II elements, Tc1/*Mariner* and *hAT* appear to be the most widespread DNA transposons, though they are not found in all taxonomic groups. In keeping with this, Wallau et al. [20] found that the rate of reported horizontal transfer of DNA transposons in animals was highest for Tc1/*Mariner* and *hAT* elements. It therefore seems likely that potential for horizontal transfer is a major factor in shaping large-scale patterns of TE distribution among eukaryotes, although differential long-term survival of TEs inherited from a distant common ancestor could also play a role in some cases.

**Table 4:** **Percentage of species found with each superfamily**

**Animals**	**Land Plants**	**Fungi**	**“Protists”**	**Eukaryotes**
*hAT* and Tc1/*Mariner* (88%)	*Gypsy* and *Copia* (100%)	*Gypsy* (87%)	*Copia* (56%)	*Gypsy* (84.05%)
CR1/L3 (78.67%)	*CMC* (95.06%)	*Copia* (77.92%)	*Gypsy* (52%)	*Copia* (75.93%)
*Gypsy* (76%)	*Mutator* + *Rehavkus* (90.12%)	Tc1/*Mariner* (68.83%)	Tc1/*Mariner* and *Mutator* + *Rehavkus* (48%)	*hAT* and Tc1/*Mariner* (69.26%)
L1 (68%)	*hAT* (88.88%)	*hAT* (40.26%)	L1 and *hAT* (44%)	*Mutator* + *Rehavkus* (54.47%)
RTE (58.67%)	PIF/*Harbinger* + ISL2EU and *Helitron* (66.25%)	*Helitron* (35.06%)	DIRS (32%)	*Helitron* (50.19%)

The top 5 percentage superfamily hits for each taxonomic group.

### Future investigations: prospects and challenges

An obvious avenue for future research will be to conduct similar analyses at finer phylogenetic scales. In the present study, comparisons were conducted across eukaryotes as well as within animals (and among vertebrates), plants, fungi, and “protists”. As more data become available, it will be very useful to compare trends (if any) within and among specific taxa. To date, however, there are insufficient data for most groups to conduct a reliable analysis with greater phylogenetic resolution.

Many of the limitations can be ameliorated by increasing the degree to which TE data are provided in genome sequence publications. Many papers reporting the results of genome or survey sequencing projects already describe TEs in an easily accessible summary table, which greatly facilitates analyses such as the one presented here. However, there is substantial variation in the level of detail provided, and in a great many cases no useable information about TE content is provided at all. In fact, half of the papers consulted could not be included in the present analysis for this reason. Of course, it is not always possible to generate fine-scale summaries of TE composition, especially for the genomes of non-model species. These limitations aside, the present analysis has highlighted some ways in which the dataset could be expanded and improved to enable further study of TE diversity and distribution.

First and foremost, a basic catalogue of TE diversity and relative abundance should be provided whenever possible. The results of such analyses are contingent on the content of the available transposable element databases (e.g., Repbase), however, so an important step will be to make a concerted effort to populate them with TEs from less well-studied genomes. In addition, automated TE detection and classification algorithms that can identify novel types of TEs, such as REPET [25] should be used more frequently, along with expert annotation, to include this information as a matter of course in future genome projects.

It is also recommended that future reports of TE diversity avoid combining functionally and phylogenetically distinct TEs into single categories. For example, some papers provide a “*Gypsy*/DIRS” category. Both of these are LTR retrotransposon superfamilies, however their replication cycle and means of integration back into the genome differ substantially, with *Gypsy* using an integrase and DIRS having a hypothesized circular intermediate and using a tyrosine recombinase [26]. Lumping such functionally disparate elements into a single category significantly reduces the possible resolution of future studies of TE diversity across genomes.

Finally, it would be very useful to increase the overall level of resolution by providing classifications below the level of superfamily where possible. Higher-order divisions such as *Copia* and *Gypsy* provide an informative first pass, but the underlying community composition of LTR elements can be phylogenetically complex, with particular sub-superfamilial groups dominating in some genomes but not in others [27]. Notably, some recent papers have begun reporting to a finer level of resolution for LTR retrotransposons in plant genomes. Descriptions of plant TE catalogs should also be more vigilant about reporting LINEs and SINEs down to the superfamily level, as these tend to be less well studied in plants.

## Conclusions

Overall, there is no straightforward relationship between eukaryotic genome size and TE diversity at the superfamily level. Instead, there appears to be an increase in TE diversity with genome size only to a certain point (specifically, around 500Mbp), and then either a lack of relationship (animals) or a negative correlation (plants) above this threshold. Variance in TE diversity is highest at mid-range genome sizes (500Mbp), and it is within this range that the highest TE diversity is observed. Larger genomes tend to contain many more copies of TEs, but these are derived from a smaller number of TE superfamilies and most copies are inactive. There are theoretical explanations that may account for these observations, but the complexity of the interacting factors means that much more work will need to be done before patterns of TE abundance, diversity, ubiquity versus taxon-specificity, and horizontal transfer can be described and understood.

## Methods

### TE diversity data

Data on TE diversity were compiled from the literature for both completed and survey-sequenced genomes (as were available up to January 2014). In total, 541 genome papers were consulted. More than half of these papers could not be included in this study because they lacked basic descriptions of TE composition. The final dataset therefore consisted of genome data for 257 species, including 75 animals, 80 land plants, 77 fungi, and 25 “protists” (including algae). Of these, 45 were from BAC-end, fosmid or survey sequencing projects and the rest were from “complete” genome sequencing projects.

Many of the relevant papers were published before the discovery of some novel superfamilies of TEs or were subject to past limitations of technology or annotation, and as such additional sources of information were searched in an effort to ensure that the TE information for each genome was as comprehensive as possible. This included searching for species-specific literature on TEs, papers characterizing novel superfamilies, and public databases such as Repbase Update, Gypsy Database, SINEbase, and taxon-specific genomic databases ([28-30]; see also Additional file 1). The superfamily level of the TE taxonomic hierarchy was chosen because it is the level most commonly reported in genome papers, and it is the best defined level of separation for TEs below that of the Class designation. The superfamilies designated in Repbase were used with some modifications: recent phylogenetic work by Yuan and Wessler [31] suggested certain separate superfamilies are grouped into well-supported clades and should be consolidated, and this convention was used here. A matrix was constructed for each species to record the presence/absence of each superfamily.

To account for novel but uncharacterized TEs, as well as TEs that remained unclassified when the original source papers were published, categories were added for the major orders of TEs (DNA transposons, LTR elements, ERVs, SINEs, LINEs, *Penelope*). These general categories were used when unknown or unclassified TEs were listed in summaries or where potentially novel superfamilies were mentioned but not well described. In total there were 75 categories detailing 69 known superfamilies and 6 unknown/unclassified categories. The taxonomic designations in this case are equivalent to superfamilies within non-LTR retrotransposon taxa [32,33].

### Genome size data

Estimates of genome size (in megabase pairs, Mbp), were obtained from the original genome papers and/or the Animal Genome Size Database [4] and Plant DNA C-values Database [5]. The raw dataset used for this analysis is provided as Additional file 1.

### Statistical analysis

Summary statistics and correlation coefficients were calculated using standard methods. However, because shared common ancestry violates the assumption of independence of species data, Felsenstein’s [34] PICs, positivized and forced through the origin, were computed using the PDAP module [35] in Mesquite v2.75 [36] whenever significant relationships were found using non-phylogenetic methods. Given the broad phylogenetic coverage of the current dataset, it was necessary to assemble phylogenetic trees manually. This was done using information provided in the Tree of Life Database [37]. These included only topology and not branch length data, so branch lengths were all set to 1 for PIC analyses. These analyses were repeated using each of the branch-length estimation methods of Grafen, Nee, and Pagel in Mesquite; there was no effect on the results in any case. In addition, one degree of freedom was subtracted for each instance of a soft polytomy [38].

Analyses were initially conducted across all available eukaryote data and, based on these results, were also performed within particular genome size ranges as well as within individual taxa (i.e., all animals, vertebrates only, land plants, and fungi; “protists” were not examined separately because this group is both undersampled and paraphyletic).

## Additional file

Additional file 1:
**TE diversity vs genome size data set.** Excel spreadsheet (.xlsx) of the data collected, analyzed and references used.

## Abbreviations

1C: Haploid genome size
ERV: Endogenous retrovirus
LINE: Long interspersed nuclear element
LTR: Long terminal repeat
Mbp: Mega base pairs
PIC: Phylogenetically independent contrast
SINE: Short interspersed nuclear element TE, transposable element
